# Genome-Wide Identification, Evolutionary Patterns, and Expression Analysis of *bZIP* Gene Family in Olive (*Olea europaea* L.)

**DOI:** 10.3390/genes11050510

**Published:** 2020-05-05

**Authors:** Siyu Rong, Zhiyang Wu, Zizhang Cheng, Shan Zhang, Huan Liu, Qianming Huang

**Affiliations:** Department of Bioresource Chemistry, College of Science, Sichuan Agricultural University, Ya’an 625000, China; 18227591075@163.com (S.R.); wuzhiyang119@163.com (Z.W.); chengxiaoyi_01@sina.com (Z.C.); zhangshanwts@163.com (S.Z.); liuhuan@163.com (H.L.)

**Keywords:** olive, bZIP, evolution, expression analysis, fruit development, lipid synthesis

## Abstract

Olive (*Olea europaea*.L) is an economically important oleaginous crop and its fruit cold-pressed oil is used for edible oil all over the world. The basic region-leucine zipper (*bZIP*) family is one of the largest transcription factors families among eukaryotic organisms; its members play vital roles in environmental signaling, stress response, plant growth, seed maturation, and fruit development. However, a comprehensive report on the *bZIP* gene family in olive is lacking. In this study, 103 *OebZIP* genes from the olive genome were identified and divided into 12 subfamilies according to their genetic relationship with 78 bZIPs of *A. thaliana*. Most *OebZIP* genes are clustered in the subgroup that has a similar gene structure and conserved motif distribution. According to the characteristics of the leucine zipper region, the dimerization characteristics of 103 OebZIP proteins were predicted. Gene duplication analyses revealed that 22 *OebZIP* genes were involved in the expansion of the *bZIP* family. To evaluate the expression patterns of *OebZIP* genes, RNA-seq data available in public databases were analyzed. The highly expressed *OebZIP* genes and several lipid synthesis genes (*LPGs*) in fruits of two varieties with different oil contents during the fast oil accumulation stage were examined via qRT-PCR. By comparing the dynamic changes of oil accumulation, *OebZIP1*, *OebZIP7*, *OebZIP22*, and *OebZIP99* were shown to have a close relationship with fruit development and lipid synthesis. Additionally, some *OebZIP* had a significant positive correlation with various *LPG* genes. This study gives insights into the structural features, evolutionary patterns, and expression analysis, laying a foundation to further reveal the function of the 103 *OebZIP* genes in olive.

## 1. Introduction

The olive (*Olea europaea* L.) is a socioeconomically important oleaginous crop that is planted widely in the Mediterranean basin, symbolizing peace, health, and longevity. The olive is the only member of the Oleaceae family with edible fruit. Olive oil is rich in unsaturated fatty acids and other important secondary metabolites. It also contains more than 30 different phenolic compounds [1] that are strong antioxidants and free radical scavengers [2]. In fact, olive oil has great benefits for human health [3], especially for cardiovascular disease, obesity, and diabetes [4]. It has also been recognized as having potential value in reducing the incidence of Alzheimer’s [5]. With the development of China’s economy, the market demand for olive oil is increasing dramatically; nevertheless, the domestic planting area is small and the production is low; therefore, consumption depends on imports. As such, it is necessary for us to reduce the cost, increase the planting yield, and improve the quality of olive oil.

The synthesis and accumulation of lipids are regulated by complex and cooperative mechanisms [6]. Transcription factors (TFs) are considered to be important factors in regulating plant development via self-regulation and regulation of downstream target gene expression. In a model plant, Arabidopsis, 8% of protein-encoding capacity is attributed to TFs [7], and more than 100 TF families have been found according to the special amino acid sequences and conserved DNA-binding domains. TFs specifically interact with cis-acting elements in the promoter or other TFs through functional domains to activate or inhibit transcription [8]. Studies have found that *MYB* [9,10], *WRI1* [11,12,13,14], *Dof* [15,16,17], and *bZIP* [18,19,20,21] play key roles in regulating lipid biosynthesis. Among them, we will focus on basic leucine zipper (*bZIP*). The plant bZIP TF contains two functional domains: a basic region and an adjacent leucine zipper. The basic region generally consists of 18 amino acid residues (aa), which is very conserved in eukaryotes [22]. The basic regions all have the same aa sequence model like N-X_7_-R/K-X_9_, which is a DNA-binding domain. The bZIP target gene sequences consist of an ACGT-core, showing palindromic-hexamers like G-boxes (CACGTG), C-boxes (GACGTC), and A-boxes (TACGTA). However, the leucine zipper region is more variable and is composed of a repeat aa sequence, L-X_6_-L-X_6_-L. This special aa arrangement makes hydrophobic amino acids all appear on one side of the *α*-helix. The result is that the leucine zipper forms an amphiphilic helical structure which is involved in the dimerization of the bZIP protein before it binds to DNA. So, bZIP TFs generally act as dimers to form a ‘scissor-grip’ model and bind dsDNA by the basic region to regulate flexibly.

With respect to function, *bZIP* genes are involved in numerous biological and physiological processes in plant growth and development [23], as well as resistance to stress [24,25]. The functional relevance of *bZIP* in numerous developmental processes is well-established, such as systemic acquired resistance [7], chloroplast development [26], pollen germination [19], stress response [27], and seed maturation, as well as oil acumination. Research on *bZIP* in seed maturation and oil acumination is particularly abundant. AtbZIP10, AtbZIP25, and AtbZIP53 interacts with ABI3 [28]. The expression of *AtbZIP53* precedes and overlaps the seed maturation gene (*MAT*). Gain- and loss-of-function approaches indicate a correlation between the amount of the bZIP53 protein and *MAT* gene expression. Arabidopsis LEC1 and L1L activate transcription by interacting with seed-specific ABRE-binding factor, bZIP67 [18]. Another study found that AtbZIP67 regulates the ω-3 fatty acid content of Arabidopsis seed oil by activating fatty acid desaturase 3 [20]. Soybean *GmbZIP123* participates in lipid accumulation in seeds, and the overexpression of *GmbZIP123* can increase the lipid content in the seeds of transgenic Arabidopsis [21].

Although the *bZIP* family has been widely studied in many plants, including *Arabidopsis thaliana* (Arabidopsis) [22], *Oryza sativa* L. (rice) [29], *Sesamum indicum* (sesame) [30], *Glycine max* (soybean) [31], *Vitis vinifera* L. (grape) [32], *Ricinus communis* L. (castor bean), *Manihot esculenta* (cassava) [33], *Fragaria vesca* (strawberry) [34], *Malus domestica* (apple) [35], and *Brassica napus* (rape) [36], there is still not enough information about it in olive. The genome of the olive was sequenced recently [37]; genome-wide data are available in the Olive Database (http://olivegenome.org). Analyzing the gene family at the genome level, we can understand the evolutionary process of the *bZIP* gene family and the differentiation of its function, so it is necessary to explore the structure and evolutionary pattern of *bZIP* at the gene family level. In this study, 103 *OebZIP* genes were identified and divided into 12 subgroups, referencing to the classification of Arabidopsis [38]. In addition, *OebZIP* genes were systematically investigated and analyzed, including chromosome distribution, gene structure, motif distribution, dimerization, gene duplication events, and evolutionary analyses with other different plant species. Furthermore, their expression analysis in different tissues (fruits, leaves, stems, pedicels) were examined using RNA-seq data which are available in public databases. To further study this matter, we selected 15 candidate *OebZIP* genes that are highly expressed in fruits based on transcriptome data. Then, we analyzed the expression profiles at different fruit development stages (July to November) of two varieties via qRT-PCR, due to the fact that only a small number of *bZIPs* have been functionally characterized in plants. Oil in olives is produced mainly in the mesocarp. The oil accumulation in the mesocarp follows a typical S-shaped curve [39]. Generally, olive oil is accumulated mostly between August and September, and reaches a plateau in November. However, due to environmental conditions, different agricultural habits, and olive varieties, the manner of lipid accumulation may be different [40]. Therefore, we preliminarily evaluate the expressive relationship with lipid synthesis genes (*LPGs*) in two varieties. We analyzed the expressive relation between *LPGs* and *OebZIP*, combined with the dynamic diversification of oil content, predicted the upstream cis-element of *LPGs* that contains A-boxes, G-Boxes, and C-Boxes, and performed a correlation analysis. Our study was the first to perform genome-wide identification and an expression analysis of *bZIP* genes. The goal is to provide a foundation to further study the functions of *OebZIP* on lipid synthesis.

## 2. Materials and Methods

### 2.1. Genome Data Sources

The olive (*Olea europaea* L.) genome (v1.0) was downloaded from the olive database (http://olivegenome.org/) [41], while the rice (*Oryza sativa* L.) (IRGSP-1.0) [42], Arabidopsis (*Arabidopsis thaliana*) (TAIR10) [43], soybean (*Glycine max*) (v2.1) [44], cacao (*Theobroma cacao*) (v2.0) [45] and grape (*Vitis vinifera* L.) genomes (12X) [46] were downloaded from the Ensembl Plants database (http://plants.ensembl.org/index.html). Sesame (*Sesamum indicum*) genome (v1.0) data were downloaded from the *Sesamum indicum* genome database (http://ocri-genomics.org) [47].

The bZIP proteins of other species were downloaded from Plant Transcription Factor Database v5.0 (http://planttfdb.cbi.pku.edu.cn/index.php).

### 2.2. Identification and Filtration

To retrieve all members of the *bZIP* gene family, we used the HMMER v3.1b2 (http://hmmer.janelia.org/) Hidden Markov model (PF00170) as a probe and to screen all the candidate proteins with E values of less than 1e-10 in order to build a new, olive-specific Hidden Markov model for the secondary search. Then, the secondary search results of the protein sequences were further confirmed using SMART (http://smart.embl-heidelberg.de/) [48] and Pfam (http://pfam.xfam.org/searc) [49] to ensure their reliability. Finally, all putative bZIP gene models were utilized to analyze the amino acid length, PI, MV by ProtParam (https://web.expasy.org/protparam/) and named by their chromosomal location.

### 2.3. Phylogenetic Analysis and Classification

In order to detect the classification and evolutionary profile of all *OebZIP*, the amino acid sequences of the Arabidopsis and olive were determined by ClustalW. The tree was constructed using MEGAX with the Maximum Likelihood (ML) method: A phylogeny test was performed with the bootstrap method of 1000 replicates, substitution with the Poisson model, and gaps Data treatment with pairwise deletion.

### 2.4. Gene Structure and Conserved Motif Analysis

We used the online tool Gene Structure Display Server (GSDS v2.0) (http://gsds.cbi.pku.edu.cn/index.php) to analyze the structure of the *OebZIP* gene containing the constituents of the exons/introns. Next, we employed MEME v 5.0.2 (http://meme-suite.org/tools/meme) to explore the conservative motifs as well as the bZIP domain, with the following optimized parameters: the maximum number of motifs was set to 10; the width of each motif was 6–50 residues.

### 2.5. Chromosomal Location, Gene Duplication, and Evolutionary Analysis

MG2C v2.0 (http://mg2c.iask.in/mg2c_v2.0/) was used to locate the *OebZIP* gene on the chromosomes of *Olea europaea*.L. Firstly, according to multiple sequence alignments, we got a number of a pairwise genes that had similar lengths and sequences by Circos (http://circos.ca/). Afterward, the MCScanX was used to find duplication genes in the olive genome and to analyze collinearity between olive and six other species (Arabidopsis, rice, sesame, soybean, cacao, and grape) [50].

### 2.6. Dimerization Properties of OebZIP Proteins

The leucine zipper region of OebZIP proteins domain, which might be related to the dimerization stability and specificity, was partitioned into different boundaries of N and C terminal [51]. To describe and speculate on the dimerization foundation of 103 OebZIP members, we set seven special amino acid residues that covered four amino acid residues before the appearance of the first leucine in the bZIP domain and the following two amino acid residues, as an initial heptad named L_0_. Next, the amino acid sequences were manually arranged to the end or the tenth heptad, named L_9_. The nomenclature of the position within each heptad was *g*, *a*, *b*, *c*, *d*, *e*, and *f*.

### 2.7. Spatiotenporal Expression Analysis of OebZIP Gene Using Transcriptome Data

To investigate the changes in expression of the *OebZIP* gene across different stages (July, November) and tissues (stem, leaf, pedicel, and fruit), we acquired olive transcriptome data [37] from the NCBI SRA database (https://www.ncbi.nlm.nih.gov/sra) (SRR4473639, SRR4473641, SRR44742, SRR4473643, SRR4473644, SRR4473645, SRR4473646, SRR4473647). After downloading the data, we used FastQC v0.11.8 to check the quality of the fastq files and trim adapters using fastp v0.19.4. Next, we mapping reads by HISAT2 v2.1.0. Counts were normalized and standardized using HTSeq v0.9.1. FPKM was calculated by Cufflinks v2.0.2 to obtain gene expression values. Finally, the heat maps were drawn using *R* v3.5.3, and the data normalization parameter was “scale = row”.

### 2.8. Plant Material

We got fresh samples from July to November in 2019 from Chengdu City, Sichuan province, China (30°45′ N, 104°32′ E, altitude of 500 ~ 600 m). Olive trees (5 years old) having a vase shape with 5 × 8 distances were randomly cultivated. Different varieties of olive, i.e., ‘Arbequina’, ‘Koroneiki’, ‘Ezhi-8’, ‘Picholine’, and ‘Grossa’, were used in this study. Each experiment was repeated at least three times.

### 2.9. Plant RNA Extraction and qRT-PCR

Total RNA was extracted using a kit (Tiangen, Beijing, China), the reverse-transcribed process was performed with a PrimeScript RT reagent kit (TaKaRa, Dalian, China), and primers were designed by Primer5. The ‘Arbequina’ and ‘Grossa’, due to the enormous difference in their oil contents, were chosen for the qRT-PCR experiment to explore the expression quantity of *OebZIPs* in leaves and fruits at fruit development stages. Then, *LPG* genes were texted. *β-Actin* was used as an internal reference gene, and the expression data were calculated by 2-(ΔΔCT) method [52].

## 3. Results

### 3.1. Identification and Naming of OebZIP

Based on a HMMER search analysis, we manually screened the candidates in two databases and got 103 *bZIP* genes in the olive genome; we named these genes according to their distribution on the chromosomes (*OebZIP1*~ *OebZIP103*, Table S1). The coding sequence length, the isoelectric points (pIs), and molecular weights (MWs) of these genes were then analyzed, as shown in Table S1. The lengths of the all *103 OebZIP* proteins were between 96 aa and 772 aa, with an average of 315 aa. The MWs of the proteins ranged from 11.22 (OebZIP99) to 84.31 (OebZIP6), with an average of 34.96. The isoelectric point (pIs) was between 4.85 (OebZIP97) and 10.99 (OebZIP96), with an average of 7.20.

### 3.2. Phylogenetic Analysis and Classification

**Figure 1:**
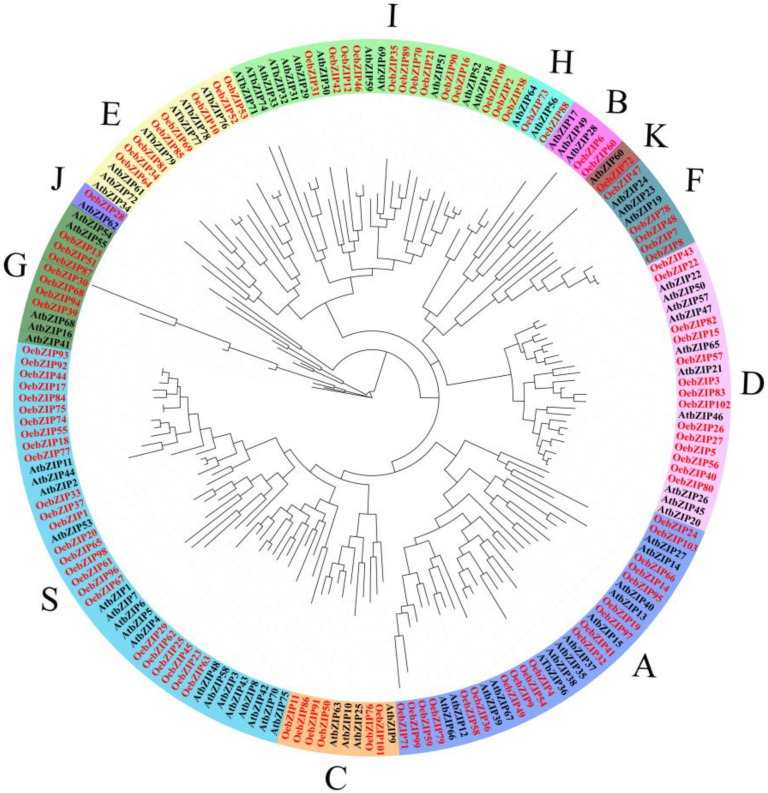
Phylogenetic tree of bZIP proteins from Arabidopsis and olive were built by the ML method. The *bZIP* genes from olive and Arabidopsis are marked in red and black, respectively. Plant bZIP members were divided into 12 subgroups (A~K, S). Different color blocks represent different subgroups. The bootstrap was set to1000.

To classify the subgroups of *OebZIPs* and explore the evolutionary relationship between the Arabidopsis and olive, we constructed an unroot phylogenetic tree based on the Maximum Likelihood (ML) method with protein sequences of 75 *AtbZIP* genes and the 103 *OebZIP* genes (Figure 1). The 75 *AtbZIP* genes were divided into 12 subgroups, with 103 *OebZIPs* being distributed in each. Similar to Arabidopsis, most of the *OebZIP* genes belong to subgroups S and A, with each containing 25 and 19 *OebZIP* genes, respectively. Interestingly, this was also observed in rice, sesame, cacao, and olive, but not grape (Figure S1). Subgroup J and K were the smallest classifications, with each including 1 *OebZIP* gene.

### 3.3. Gene Structure and Motif Composition of Olive bZIP

**Figure 2:**
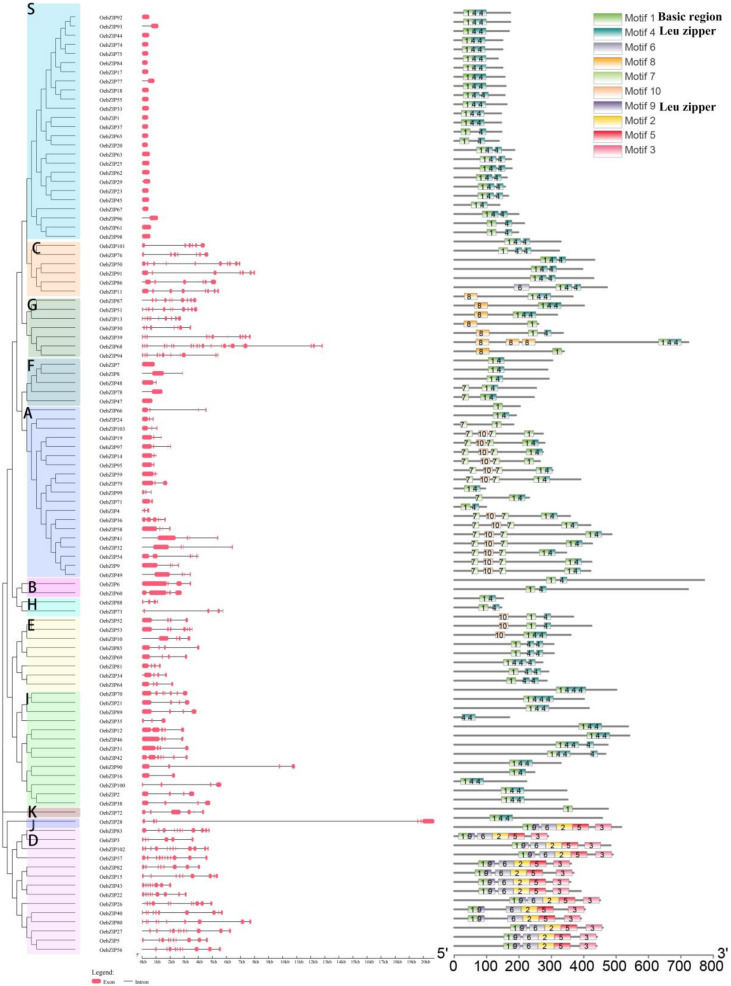
Phylogenetic tree. The distribution of the conserved protein motifs and gene structures of *OebZIP* genes. Different color blocks represent different subgroups. Red boxes indicate exon region, and black lines indicate intron. The motifs are numbered 1–10 and displayed in boxes of different colors. Motif 1 indicates the basic region of bZIP domain and motifs 4 and 9 indicate a Leu zipper of the bZIP domain. Detailed sequence information for each motif is shown in Figure S2.

As shown in Figure 2, the intron/exon structure was detected using the Gene Structure Display Server (http://gsds.cbi.pku.edu.cn/index.php/). The number of exons of *OebZIP* genes varied from 1 to 22, of which 22 (21.4%) of the 103 *OebZIP* genes had only one exon, and most of them were fastened to subgroup S. Subgroups C, D, and G had multiple exons: 5 genes had a number of exons ranging from 6 to 10, 14 had between 6 to 12 exons, and 7 had between 8 to 22.

MEME 7.0 was used to detect the conserved motifs in 103 OebZIP proteins, and 10 motifs containing the bZIP domain were identified. As shown in Figure 2, most OebZIP proteins contained several motifs, while OebZIP72, OebZIP94, OebZIP66, OebZIP103, OebZIP19, and OebZIP95 only contain motif 1, which means that only the basic region has no obvious Leu zipper structure. OebZIP35 only contains motif 4, which only has a Leu zipper region and lacks the Basic region. Motif 8 is highly conserved and only exists in subgroup G, indicating that it is a conserved protein domain which may perform a special function. In subgroup A, except for OebZIP4, OebZIP24, and OebZIP99, all the others contained motifs 7 and 10. The distribution of the protein sequence was motif 7–motif 10–motif 7. OebZIP in Subgroup D contained the most motifs among the 13 subgroups, and was arranged in the order of motif 1–motif 9–motif 6–motif 2–motif 5–motif 3; among these, motifs 9, 2, 5 and 3 are unique in subgroup D. In addition, the evolutionary tree clustering results are consistent with the intron/exon distribution and motif distribution. This indicated that most of OebZIP genes in the same subgroup showed low structural diversity, close evolutionary relationships and high degrees of conservation.

### 3.4. Chromosomal Locations and Gene Collinearity Analysis of OebZIP

**Figure 3:**
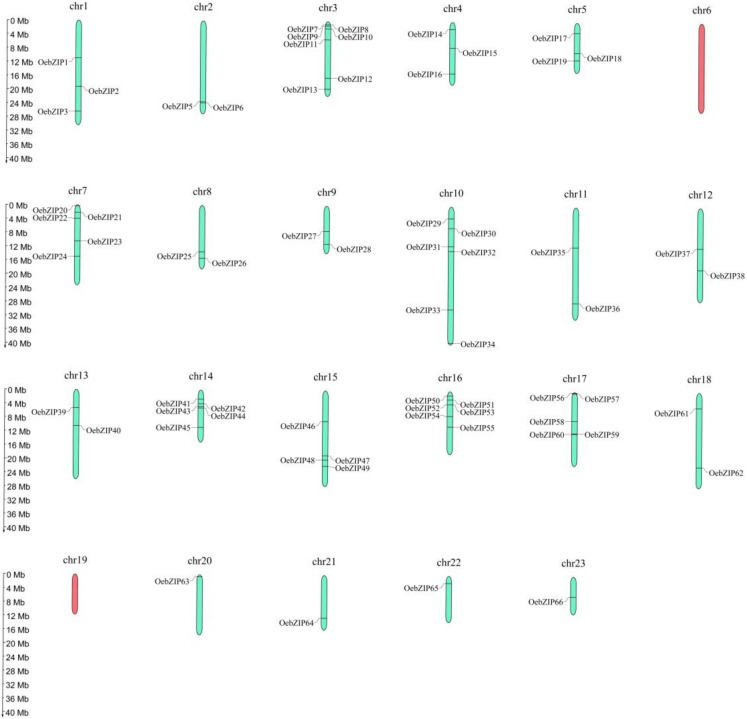
The distribution of *bZIP* genes on olive chromosomes. The red color represents chromosomes without *OebZIP* genes and the green is the chromosome that has *OebZIP* genes. The relative length of the chromosomes is millions of base pairs (Mb).

Sixty-seven *OebZIP* gene locations were mapped on the chromosome (Figure 3). Thirty-six *OebZIP* locations were not distributed on the chromosome but rather, on scaffolds, and no *OebZIP* genes were distributed on chr6 and chr19. In some specific regions of chr3 and chr16, *OebZIP* genes were densely distributed.

**Figure 4:**
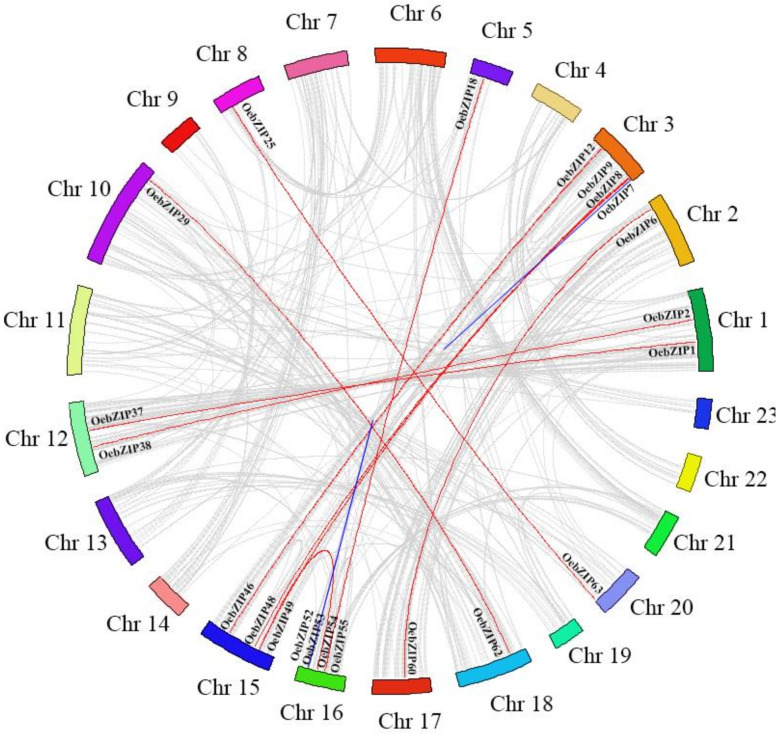
Gene duplication events in the *OebZIP* gene family. The *OebZIP* genes linked by the red line are the segment repeats, while the *OebZIP* genes linked by the blue line are the tandem repeats. Different color blocks represent different chromosomes.

Gene replication is considered to be one of the main drivers of the evolution of the genome and genetic systems. Segment repeats and tandem repeats are the two main reasons for the expansion of plant gene families. In the olive genome, there were four pairs of tandem repeat events involved in 8 *OebZIP* genes; *OebZIP7* and *OebZIP8*, and *OebZIP52* and *OebZIP53* were distributed on chr3 and chr16, respectively, while *OebZIP74* and *OebZIP75*, and *OebZIP92* and *OebZIP93* were not distributed on the chromosome. As such, they were not represented graphically in Figure 4. Twenty-seven *OebZIP* genes were involved in 14 fragment repeats. A pair of genes that formed one gene duplication event was found to come from the same subgroup. As *OebZIP7* and *OebZIP8* are tandem genes, they were both assigned to subgroup F. Thus, 33.98% of the *OebZIP* genes were shown to be involved in gene duplication, which may be why so many OebZIP genes are caused by gene duplication.

**Figure 5:**
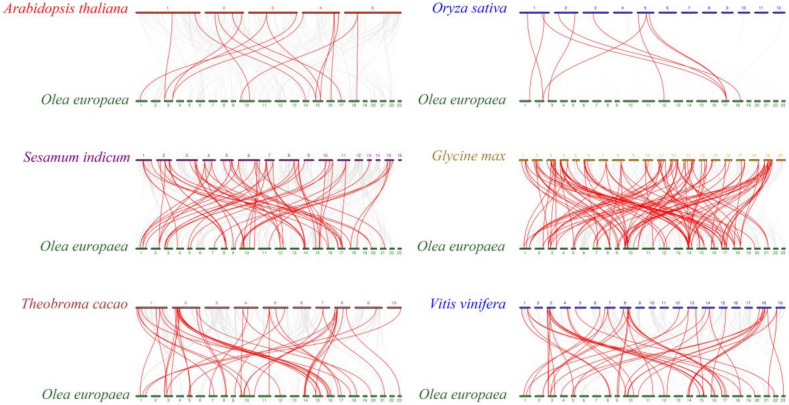
Synteny analysis of *OebZIP* with other species (*Arabidopsis thaliana*, *Sesamum indicum*, *Glycine max*, *Vitis vinifera* L., *Theobroma cacao,* and *Oryza sativa* L.). Gray lines represent collinear blocks within olive and other species, while red lines represent syntenic *bZIP* gene pairs.

To explore the evolutionary relationship of *bZIP* genes in different species, we used McScanX to perform a gene collinearity analysis on five dicotyledonous plants: *Arabidopsis thaliana*, *Sesamum indicum*, *Glycine max*, *Vitis vinifera* L., and *Theobroma cacao*; and on a monocotyledon: *Oryza sativa* L. (Figure 5). Many reference plant *bZIP* genes had a colinear relationship with some *OebZIP* genes, i.e., 14 (Arabidopsis), 9 (rice), 38 (grape), 35 (cacao), 81 (soybean) and 51 (sesame). Soybean had the most *bZIP* genes that were colinear with olive *bZIP* genes, followed by sesame; rice had the fewest. In addition, 5 *OebZIP* genes (*OebZIP1*, *OebZIP29*, *OebZIP46*, *OebZIP33*, *OebZIP44*) were found to be colinear with five dicotyledons, and except for *OebZIP46*, the rest were all in subgroup S, indicating that these genes are highly conserved during evolution and play an important role in the amplification of the *bZIP* gene family in olive.

### 3.5. Prediction of Dimerization Properties

The structural characteristics of the Leu zipper region is L-X_6_-L-X_6_-L. Hydrophobic and electrostatic interactions mediate *α*-helical oligomerization to form homo- or heterologous leucine zipper structures which are involved in the bZIP protein dimerization before binding to DNA. Studies have demonstrated that every seven amino acid residues of the leucine zipper region are set as a heptad, and that the arrangement order is *g*, *a*, *b*, *c*, *d*, *e,* and *f*. The possibility, stability, and specificity of the leucine zipper dimerization structure are mainly determined by four sites: *a*, *d*, *e*, and *g*. The *a* and *d* positions are usually hydrophobic amino acids. The two monomer *α*-helical structures interact with each other through the hydrophobic amino acids at the *a* and *d* positions to form a hydrophobic inner core, enabling the dimer to exist stably. Asparagine at the *a* position can form a polar pocket on the hydrophobic interface. When *aʹ* is asparagine, it can form a very stable N-N structure (*a*↔*a′*), so it tends not to interact with other amino acids, which limits the possibility of interactions between heterologous helices. The *d* position is generally a highly conserved leucine, which is one of the important factors affecting the dimerization structure. However, the *e* and *g* positions that flank the dimerization interface frequently contain charged amino acids including acidic amino acids glutamic acid (E) and aspartic acid (D), and the basic amino acids arginine (R) and lysine (K). As a result, salt bridges are formed to maintain stability between the spirals.

We performed a detailed dimerization analysis to characterize the amino acids present at positions *a*, *d*, *e*, and *g* of the OebZIP proteins (Figure S3). The length of the Leu zipper in the OebZIP family varied from two to nine heptads. At the *a* position, asparagine (Asn/N) accounted for 19%; meanwhile, at position *a*, the asparagine appeared most frequently in the second heptad(L_2_), followed by L_4_, accounting for 35.35% and 25.25%, respectively. In addition, at position *a*, the frequency of hydrophobic amino acids (I, V, L, M) was 35%, and charged amino acids (R, K, E, D) was 20%. At the *d* position, the frequency of hydrophobic amino acids was 81%, of which 50% was leucine and 31% was other hydrophobic amino acids (I, V, M).

**Figure 6:**
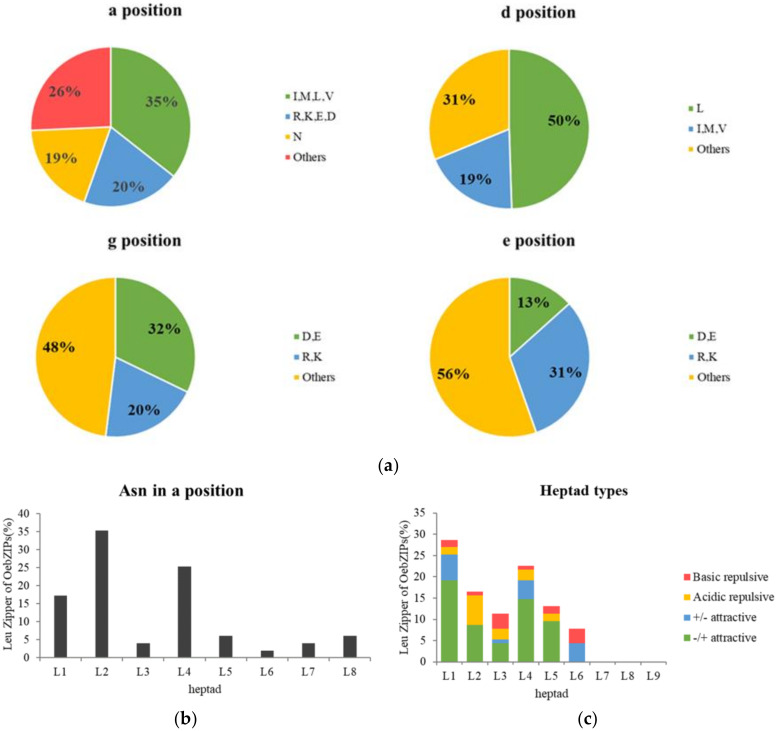
Prediction of the dimerization properties of OebZIP proteins. (**a**) Pie charts depicting Figure 3 for the amino acid positions within the Leu zipper regions. (**b**) Histogram of the frequency of Asn residues present at the position of each heptad within the Leu zippers for the OebZIP proteins. (**c**) Histogram of the frequency of attractive or repulsive g↔e′ pairs per heptad within Leu zippers for OebZIP proteins.

Four types of *g*↔*e′* interactions in each heptad were then analyzed, including attractive basic–acidic pairs (+/− attractive), attractive acidic–basic pairs (−/+ attractive), repulsive basic pairs, and repulsive acidic pairs (Figure 6). As shown in Figure 6c, the L_1_ heptad had a total of 32 of *g*↔*e′* pairs, of which 87.88% were attractive pairs. The L_4_ and L_5_ heptad had 26 and 15 *g*↔*e′* pairs respectively. The L_6_ heptad only had attractive basic–acidic pairs (+/-). The L_7_, L_8_, and L_9_ heptad had no complete *g*↔*e′* pair.

Based on the dimerization features, OebZIP proteins were divided into three categories: (I) Those that tend to form homodimerization. OebZIP proteins in this category contained the *g*↔*e*′ pairs and the asparagine in the *a* position. The members of subgroup A basically belong to this category; (II) Those with both homo- and heterodimerization. Most OebZIP belong to this category, such as those of subgroup C, which contained repulsive *g*↔*e*′ pairs and attractive *g*↔*e*′ pairs, or contain repulsive *g*↔*e*′ pairs. (III) Those that tend to form heterodimerization. OebZIP proteins in this category contained repulsive *g*↔*e*′ pairs only; for example, OebZIP30 contained three adjacent repulsive *g*↔*e*′ pairs, but there were fewer OebZIPs in this category.

### 3.6. Gene Expression profiles of OebZIPs

#### 3.6.1. Gene Expression Profiles of OebZIPs on Transcription Data

We analyzed the expression profiles based on transcriptome data including different periods and tissues. In July, 92, 95, 97, and 94 *OebZIP* genes were expressed, respectively, in fruits, pedicels, stems, and leaves; In November, 89, 96, 95, and 93 *OebZIP* genes were expressed, respectively, in fruits, pedicels, stems, and leaves. Among them, only one *OebZIP* gene (*OebZIP33*) was not expressed in four tissues in two periods, while the rest were expressed in at least one tissue. However, the gene expression abundance was significantly different. As shown in Figure S4, expression of most *OebZIP* genes was tissue-specific. For example, the *OebZIP84* gene was expressed in fruits only, while the *OebZIP62* gene was expressed in all tissues except for fruits. Moreover, most *OebZIP* genes were significantly up- or down- regulated in different periods. The highly expressed *OebZIP* genes in fruits may be involved in fruit oil accumulation, and there were 17 and 16 *bZIP* genes with higher expression in fruit in July (*OebZIP1*, *7*, *8*, *10*, *12*, *22*, *27*, *37*, *50*, *52*, *53*, *78*, *84*, *85*, *91*, *94*, and *99*) and November (*OebZIP22*, *39*, *46*, *48*, *50*, *52*, *53*, *59*, *65*, *70*, *73*, *79*, *84*, *92*, *93*, and *99*), respectively. A total of 5 *OebZIP* genes (*OebZIP53*, *99*, *22*, *50*, and *52*) were found to be highly expressed in both periods; 12 *OebZIP* genes were upregulated and 15 *OebZIP* genes were downregulated. Therefore, 15 candidate *OebZIP* genes that possess higher expression in fruits were selected for the next analysis.

#### 3.6.2. Expression Patterns of OebZIP Genes in Different Tissues

To give an insight into the functional role of the *OebZIP* gene, we used qRT-PCR to detect the expression profiles of candidate *OebZIP* genes throughout the development of fruit, and the expression of 15 candidate *OebZIP* genes in the leaf and fruit of ‘Arbequina’ were analyzed (Figure S5). All candidate genes were expressed in both fruits and leaves, but the expression levels were significantly different between different tissues at various development periods. Similar to the result of the transcriptome data analysis, most of the *OebZIP* genes had high expression in fruits. The *OebZIP22*, *OebZIP53*, *OebZIP79*, *OebZIP84*, *OebZIP91*, and *OebZIP99* had higher expression abundance in fruits than leaves in all periods (July to November).

#### 3.6.3. Expression of OebZIP Genes in Different Varieties During Fruit Development

**Figure 7:**
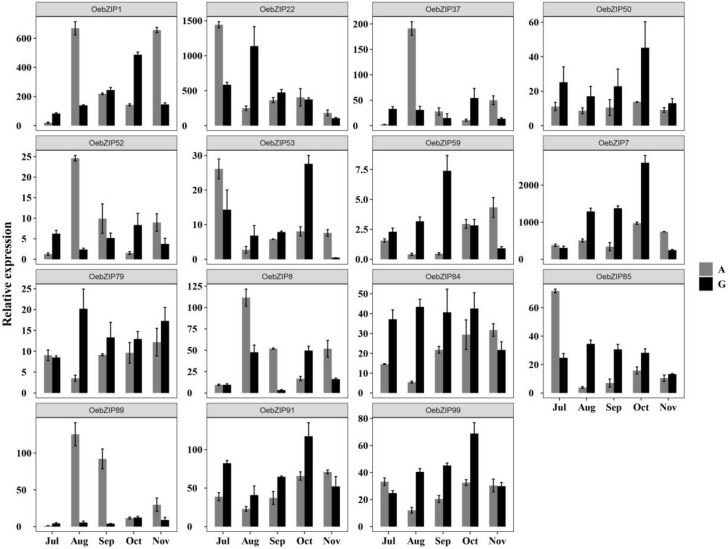
The expression level of 15 *OebZIP* genes in different varieties during fruit development. The expression level of 15 *OebZIP* genes in fruit of different varieties from July to November were examined by qRT-PCR. The A indicates ‘Arbequina’, and the G indicates ‘Grossa’. The error bars were obtained from the mean values of three replicates ± standard deviation (SD).

We found that different varieties of olive displayed a big difference in the speed of oil accumulation during the fast oil accumulation stages. The fruit development and oil accumulation period of some varieties were significantly earlier than those of others. Therefore, ‘Arbequina’ (‘A’) and ‘Grossa’ (‘G’) were selected for the real-time PCR, and to explore the candidate *OebZIP* genes among two varieties. As shown in Figure 7, the expression of candidate genes between the two varieties (‘A’ and ‘G’) was quite different. We found that some *OebZIP* genes (*OebZIP1*, *OebZIP22*, *OebZIP37*, *OebZIP52*, *OebZIP53*, *OebZIP8*, *OebZIP85,* and *OebZIP89*) in ‘A’ were concentrated in the early stages of oil accumulation (July to August). Seven *OebZIP* genes (*OebZIP1*, *OebZIP7*, *OebZIP50*, *OebZIP53*, *OebZIP59*, *OebZIP91*, and *OebZIP99*) in variety ‘G’ were concentrated in the middle stages (September to October) of oil accumulation. Furthermore, the expression levels of five *OebZIP* genes (*OebZIP1*, *OebZIP7*, *OebZIP59*, *OebZIP84*, *OebZIP85,* and *OebZIP85*) in variety ‘G’ increased dramatically from July to October, reached a peak in September or October, and then decreased rapidly. This observation is similar to the rate of oil accumulation in ‘G’, varieties indicating that these *OebZIP* genes have tight functions in olive fruit development and lipid synthesis. Interestingly, the expression level of the *OebZIP22* gene in ‘A’ in July was the same as that of the *OebZIP22* gene in ‘G’ in August, the expression levels and dynamic changes of the two varieties were again similar, indicating that *OebZIP22* gene may play an important role in fruit development, given the different fruit development periods of the two varieties.

In addition, although two pairs of tandem genes (*OebZIP7* & *OebZIP8*, *OebZIP52,* and *OebZIP53*) and one pair of fragment genes (*OebZIP1* and *OebZIP37*) had sequence similarities, their expression patterns and abundances were disparate, indicating that the repeated genes in olive achieved functional differentiation during their evolution.

### 3.7. Predicting Interaction with Lipid Synthesis Genes

Since the expression of some *OebZIP* genes was found to be significantly different between the two varieties, these genes might correlate with oil accumulation. To preliminarily investigate this interaction, we referred to previous studies and scanned 44 lipid synthesis genes (*LPGs*) named according to their chromosomal location from the olive genome (Table S2).

#### 3.7.1. Expression Patterns of Lipid Synthesis Genes

**Figure 8:**
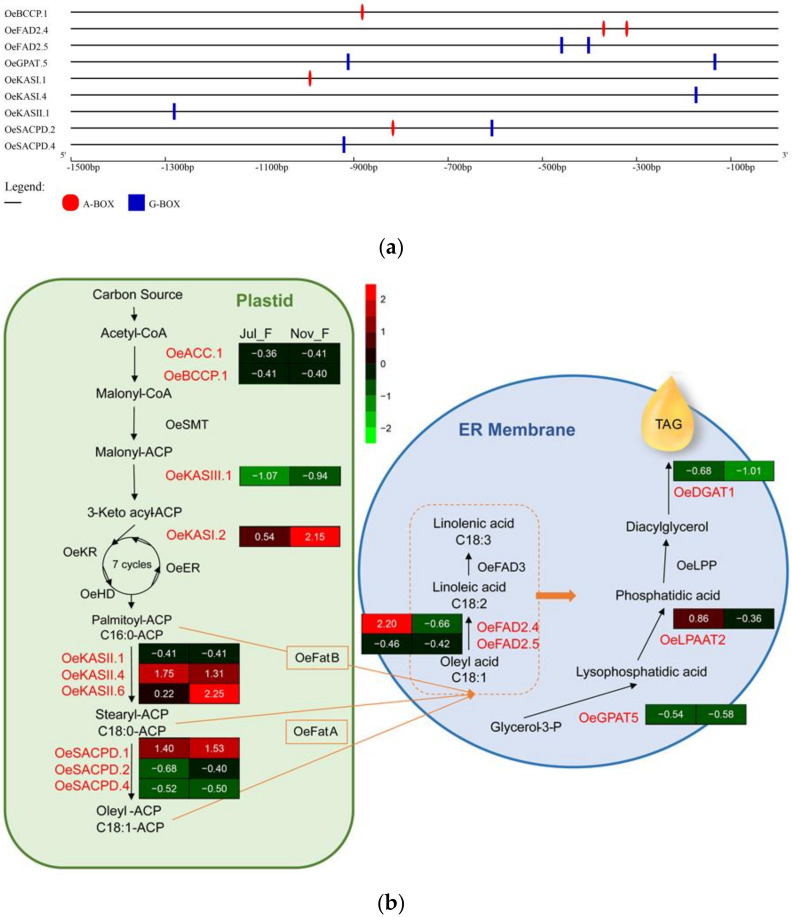
The prediction of upstream cis-acting elements of *LPG* genes and the triacylglycerol (TAG) biosynthesis pathway in olive. (**a**) The promoter sequences (−1500 bp) of 9 LPG genes were analyzed. The elements were identified as follows: red for the A-box element (TACGTA) and blue for the G-box (CACGTG) element. The scale at the bottom indicates the number of the nucleotides to the translation initiation codon. (**b**) Red text represents the key enzymes involved in the study, while black represents those not involved. ACC: acetyl-CoA carboxylase; BCCP: biotin carboxyl carrier protein; SMT: S-malonyl-transferase; KAS: *β*-ketoacyl-ACP synthase; KR: 3-ketobutyryl-ACP reductase; HD: 3-hydroxybutyril-ACP dehydratase; ER: enoyl-ACP reductase; FatA: fatty acid-ACP thioesterase A; FatB: fatty acid-ACP thioesterase B; SACPD: stearoyl-ACP desaturase; FAD: fatty-acid desaturase; GPAT: glycerol-3-phosphateacyltransferase; LPAAT: lysophosphatidate acyltransferase; LPP: phosphatidate phosphohydrolase; DGAT: diacylglycerol acyltransferase. Green indicates low expression, dark indicates intermediate expression, and red indicates high expression. The numbers in the boxes indicate normalized scale.

Based on the abundance of genes expression (Figure S7) and the prediction of upstream cis-acting elements (Figure 8a), 15 genes (*OeACC1*, *OeBCCP2, OeDGAT1*, *OeFAD2.4*, *OeFAD2.4*, *OeGPAT5*, *OeKASI.2*, *OeKASII.1*, *OeKASII.4*, *OeKASII.6*, *OeKASIII.1*, *OeLPPAT2*, *OeSACPD1*, *OeSACPD2*, and *OeSADCP4*) were selected. These basically covered most of the genes involved in the lipid synthesis pathway (Figure 8b); therefore, these genes were selected for detailed qRT-PCR analysis.

**Figure 9:**
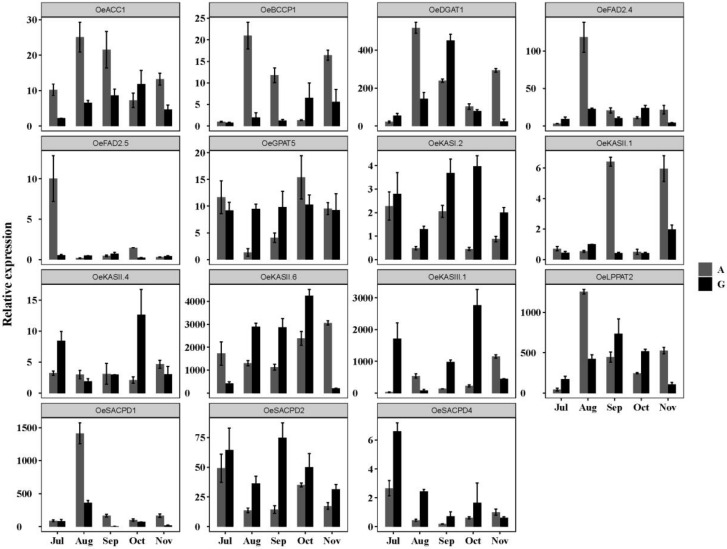
Expression levels of 15 *LPG* genes during fruit development in different varieties. qRT-PCR was used to detect the expression level of the *LPG* gene in fruits of different varieties from July to November. A represents ‘Arbequina’, and G represents ‘Grossa’. The error bars indicate the mean ± standard deviation (SD) of three replicates.

The expression patterns between *LPGs* were significantly different (Figure 9). *OeKASII.6* (*β*-ketoacyl -ACP synthetase II, C16~C18), *OeKASIII.1* (*β*-ketoacyl-ACP synthetase III, C2~C4), *OeLPAAT2* (lysophosphatidate acyltransferase) and *OeDGAT1* (diacylglycerol acyltransferase) had higher expression abundance than the other *LPG* genes in both varieties. Moreover, the expression patterns of different members in the gene family were also significantly different. For example, the *OeKASII* and *OeSACPD* families had different expression patterns. Some genes (*OeFAD2.5*, *OeSACPD4*, and *OeKASII.4*) maintained an extremely low level of expression in two varieties, indicating that they may be less correlated with the regulation of fruit development and lipid synthesis. In addition, several *LPGs* had different expression patterns between the two varieties, which might be caused by the difference in the period of oil accumulation, as described above. There were 7 genes in ‘A’ (*OeACC1*, *OeBCCP1*, *OeDGAT1*, *OeFAD2.4*, *OeFAD2.5*, *OeLPPAT2*, and *OeSACPD1*) that were significantly expressed in the early stage (July to August). In contrast, in ‘G’ there were 6 genes (*OeACC1*, *OeBCCP1*, *OeDGAT1*, *OeGPAT5*, *OeKASII.6*, *OeLPPAT2*) that were upregulated progressively throughout the oil accumulation until they reached a peak in the middle stages of oil accumulation (September to October), and then downregulated rapidly. Compared with the expression patterns of *OebZIPs* and *LPGs*, the result indicates that some *OebZIPs* (*OebZIP1*, *OebZIP7*, *OebZIP91*, and *OebZIP99*) and *LPGs* (*OeACC1*, *OeDGAT1*, *OeLPPAT2*, and *OeKASII.6*) showed similar expression patterns in different varieties.

#### 3.7.2. Correlation Analysis of OebZIP with Lipid Synthesis Genes

**Figure 10:**
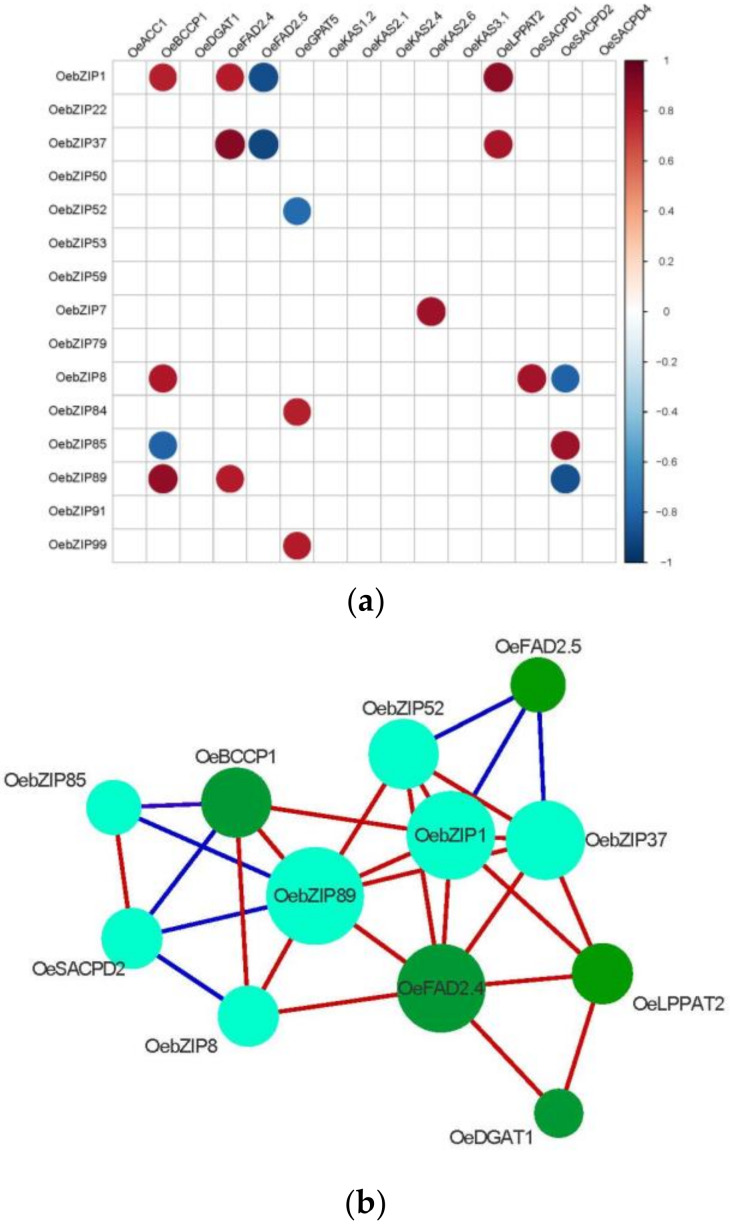
Correlation analysis of *OebZIP* with lipid synthesis genes (**a**) Red dot indicates a positive correlation; blue dot indicates a negative correlation, while the different colors represent the *p*-values of the correlation. (**b**) The predicted interaction network of *OeLPGs* and *OebZIPs*. Red lines are positive regulations, blue lines are negative.

Based on qRT-PCR experiment data of the two varieties, a correlation analysis by *R* is summarized in Figure 10. We determined that 7 *OebZIPs* had a significant positive correlation with various *LPG* genes (*OebZIP1* and *OeBCCP1*/*OeFAD2.4*/*OeLPPAT2*; *OebZIP7* and *OeKASII.6*; *OebZIP8* and *OeBCCP1*/*OeSACPD1*; *OebZIP37* and *OeFAD2.4*/*OeLPPAT2*; *OebZIP84* and *OeGPAT5*; *OebZIP85* and *OeSAD2*; *OebZIP89* and *OeBCCP1*/*OeFAD2.4*; *OebZIP99* and *OeGPAT5*). In contrast, 6 *OebZIP* genes had a significant negative correlation with *LPG* genes (*OebZIP1* and *OeFAD2.5*; *OebZIP37* and *OeFAD2.5*; *OebZIP52* and *OeGPAT5*; *OebZIP8* and *OeSAD2*; *OebZIP85* and *OeBCCP1*; *OebZIP89* and *OeSAD2*).

## 4. Discussion

### 4.1. Evolutionary Analysis of the OebZIP Gene Family

Olive is a very important oil crop around the world. With the completion of olive genome sequencing and the rapid development of bioinformatics, the mining and identification of gene families based on genome-wide searches have become more and more effective. Therefore, it is necessary to explore the structural and evolutionary characteristics of *OebZIP* genes from the level of gene families. Although the *bZIP* gene family has been studied in many plants, this is not the case for olive. In this study, a total of 103 *OebZIP* genes were identified and screened based on the olive genome. The average length of OebZIP proteins is 315 aa. With respect to numbers and aa length, compared to other plants, Arabidopsis (78, 321 aa) [38], rice (89, 311 aa) [29], sesame (63, 339 aa) [30], soybean (160, 343 aa) [31], grape (47, 336 aa) [32], and apple (114, 411 aa) [35], the number of *bZIP* family members in olive is larger and similar to apple, while the coding sequence length of the genes is clearly different. Several studies [53,54] have found that there is no positive correlation between the numbers of *bZIP* genes and the genome size. The differences in the number of *bZIP* members in plants might be due to whole-genome duplication (WGD) events [37]. Phylogenetic tree analysis with the Arabidopsis bZIP protein, which was divided into 12 subgroups, showed that the *AtbZIP* genes have a close kinship with the *OebZIP* genes. The number of identified *OebZIP* genes in the subgroups were similar to other plants (Arabidopsis, rice, sesame, soybean, and cocoa) except grape. Additionally, we found that subgroup H contains 2 *OebZIP* genes, similar to Arabidopsis bZIP. This indicates strong homology, and that subgroup H (*OebZIP73*, *OebZIP88*) may play an important role in photomorphogenesis.

Gene replication is considered to be one of the main drivers of genetic system evolution. The two main reasons for the expansion of plant gene families are tandem repeats and segment repeats. Twenty-two olive, 119 soybean, 8 tartary buckwheat, and 28 grape *bZIP* genes are involved in gene duplication events (Figure 4), accounting for around 21.4% (olive), 86.2% (soybean) [31], 8.3% (tartary buckwheat) [54], 59.6% (grape) [32]. The contribution of gene duplication events to the amplification of gene families was significantly different among different species. In addition, by comparing the quantity of the tandem repeats, i.e., 4 pairs of olive, 1 pair of soybean [31], 2 pairs of tartary buckwheat [54], 0 pairs of grape, we concluded that the contribution of tandem repeats during the evolution of *OebZIP* genes was limited, which is consistent with reports on grape, soybean, and tartary buckwheat. Therefore, segment gene duplication plays an important role in *bZIP* gene family expansion in olive. Soybean had the most *bZIP* genes that were colinear with olive *bZIP* genes (Figure 5) and tartary buckwheat *bZIP* genes [54], which may be due to the large genome of soybean and the large number of *GmbZIP* genes.

Most *OebZIP* genes in the same subgroup possess the same features (Figure 2), as also reported for apple [35], melon [55], and cassava [33], i.e., the *OebZIP* genes in each S subgroup have one exon and the motif distribution is simple, just containing motif 1 (basic area) and motif 4 (Leu area). Some specific motifs (motif7, motif10) only exist in a specific subgroup (subgroup A). Meanwhile, the order of motif arrangement in the same subgroup is consistent. Furthermore, motif 8 was found to be quite conserved and focused on subgroup G, indicating this motif might have some specific function.

The function of bZIP proteins requires dimerization. We investigated the dimerization patterns of OebZIP proteins, which are characterized mainly by the four amino acids presenting at the *a*, *d*, *g*, and *e* positions (Figure 6). At the *d* position, the frequency of Leu aa accounts for 50%, which is close to AtbZIPs (56%), but significantly less than in OsbZIPs (71%) [29], ZmbZIPs (70%) [56], and GmbZIPs (68%) [31]. The frequency of Asn at position *a* was 19%, which was lower than that found in AtbZIP (40%) [51] and GmbZIP (25%) [31]. Another difference is that in the olive, the frequency of Asn at the *a* position was the highest in the L_2_ heptad, followed by the L_4_ heptad, while in [31] Arabidopsis [51] and strawberry [34], the frequency of Asn in the *a* position was highest in the L_2_ heptad, followed by the L_5_ heptad. Therefore, it was shown that the bZIPs in olive are more prone to heterodimerization, and the length of Leu zipper is shorter compared to soybean [31], Arabidopsis [51], and strawberry [34].

### 4.2. The Expression Pattern of OebZIP was Related to Fruit Development and Lipid Synthesis

Plants have a large number of *bZIP* genes and diverse functions, but investigations of *bZIP* functions have been mainly focused on some model plants [38]. Many study already found that the *bZIP* gene family plays a role in all stages of plant growth and response to biotic/abiotic stresses [18,19,20,24,25,26,28,57,58,59,60,61,62,63]. However, very few *bZIP* genes involved in fruit development and lipid synthesis have been identified in plants, especially in olive. We explored the possibility of the involvement of candidate *OebZIP* genes in regulating lipid synthesis and gene expression levels of *LPG* genes during fruit development. In a previous study, the oil content of different olive varieties was significantly differentiated in the fast oil accumulation stage (Figure S6). Several *LPGs* have different expression patterns between the two varieties; this result is similar to that of a study on the gene expression levels of *OeFAD2* [64], *OeFAD3* [65], and *OeSACPD* [66] during fruit development between two olive varieties (‘Picual’ and ‘Arbequina’). The expression levels of some *LPGs*, such as *OeACC1*, *OeDGAT1*, *OeKASII.6*, and *OeLPAAT2*, were consistent with the oil accumulation trend. The oil in ‘A’ accumulated mainly in early stage fruit development, while the oil ‘G’ accumulated mainly in the late stage. The gene expression pattern of *OeDGAT1* in ‘G’ was consistent with a previous report of *OeDGAT1* in ‘Koroneiki’ [67], presenting a typical normal curve, i.e., following maximal mRNA levels in September and then declining substantially. Although most TAGs in olive accumulated before they matured, it has been demonstrated that some oil cultivars, like ‘A’ in our study, have much shorter oil-filling periods than ‘Koroneiki’ and ‘G’ [68,69,70] (Figure S6). Olive oil is known for its high oleic acid content. Several researchers have reported that the composition of olive oil fatty acids, particularly oleic acid, fluctuated according to the variety [68,71,72]. The variation of *OeFAD2* [64,73], *OeFAD3* [65], and *OeSACPD* [66,74] gene expression is the main factor that affects the composition of fatty acids [37]. The gene expression levels of *OeSACPD1* and -*2* in ‘A’ in our study were similar to those in a previous report in the same variety, peaking in the early stages (July to August) and then decreasing [66]. Moreover, the expression level of *OeFAD2*.*2*, which is considered to be mainly responsible for the linoleic acid content in olive oil, as reported by Hernandez [64] and Bruno [73], was higher than that of *OeFAD2.5* in our study, indicating that *OeFAD2*.*5* may not be a significant factor affecting the oil content of mesocarp.

The goal of this study was to obtain more insight into the expression patterns and functions of *OebZIP* genes during fruit development and lipid synthesis. An expression profile analysis indicated that *OebZIP* genes expression showed significant variation among different tissues, which implied that they might have diverse functions. It is therefore hypothesized that highly expressed genes in fruits are closely related to fruit development and lipid synthesis. In previous studies, some candidate *OebZIPs* expressed in fruits showed different expressive patterns in the two varieties. In addition, paired gene duplication events showed different expression patterns, suggesting that functional differences had occurred during evolution, as similarly demonstrated in previous reports on rice and grape.

Only four genes, i.e., *OebZIP1*, *OebZIP7*, *OebZIP91,* and *OebZIP99,* were significantly upregulated in the fast oil accumulation stage between the two varieties. In Arabidopsis, *AtbZIP53* was reported to be involved in regulating seed development, and seed maturation was affected when *AtbZIP53* [28] was overexpressed or knocked out. In olive, *OebZIP1* was homologous to *AtbZIP* genes, and *OebZIP1* was clustered into the same group with *AtbZIP53* in the phylogenetic tree (Figure 1). Therefore, we speculate that the *OebZIP1* may also regulate olive fruit development and oil accumulation. In addition, *OebZIP22* was found to have a similar expression pattern between the two varieties, but the expression of *OebZIP22* in ‘A’ took precedence over that of ‘G’. *OebZIP22* is a homolog of *TGA3* (*AtbZIP22*). In Arabidopsis, *TGA3* was involved in mediating hormonal cross-talk between salicylic acid and cytokinin [75]. Therefore, *OebZIP22* may be involved in hormone regulation in fruit development and lipid synthesis. In addition, through a correlation analysis, we found that the expression patterns of some *OebZIP* genes in fruits of different varieties were significantly correlated with oil synthesis genes (Figure 10). For example, there was a significant positive correlation between the expression of *OebZIP1*, *OebZIP37*, *OebZIP 89,* and *OeFAD2.4*. In addition, there were two a-boxes upstream of the *OeFAD2.4* promoter region, and the possible interactions between *OeFAD2.4* and OebZIP1, 37, and 89 transcription factors might be the focus of future research. The functions of these *OebZIP* genes need to be verified by experiments in the future.

## 5. Conclusions

In this study, for the first time, we identified 103 *bZIP* genes based on the olive genome, which were divided into 12 subgroups. We analyzed the genetic structure, conserved domain distribution, the dimerization feature, the evolutionary relationship and the gene replication events. Meanwhile, some important candidates of *OebZIP* genes for olive fruit development and lipid synthesis were identified. This study laid the foundation for the study of the structure and function of the *bZIP* gene family in olive. It provides important clues for increasing the oil content of olive from the direction of bZIP TF regulation of lipid synthesis.

## Supplementary Materials

Supplementary Materials can be found at https://www.mdpi.com/2073-4425/11/5/510/s1, Figure S1: Classification of *bZIP* genes in six plants, Figure S2: A detailed motif introduction for all OebZIP proteins, Figure S3: Amino acid sequences of the Leu zipper region of *OebZIP* proteins, Figure S4: Heatmap of 103 *OebZIP* genes expressed among four tissues based on transcription data, Figure S5: Relative expression of *OebZIP* genes in fruit and leaf, Figure S6: Dried fruit oil content during the fast oil accumulation stages (July to November), Figure S7: Heatmap of 44 *LPG* genes expressed among four tissues based on transcription data. Table S1: The basic characteristics of olive *bZIP* gene family, Table S2: The chromosomal location of olive *LPG* genes.
