# Supplementary material for: Genome-Wide Identification, Evolutionary Patterns, and Expression Analysis of bZIP Gene Family in Olive (Olea europaea L.)
Source: Genes (Basel). 2020 May 5;11(5):510. doi: 10.3390/genes11050510 (PMC7288668; doi:10.3390/genes11050510)
Supplement: Supplementary file 1 [file genes-11-00510-s001.pdf]

Supplementary

Table 1S. The basic characteristics of olive *bZIP* gene family

Gene name	Sequence ID	Chromosome	Subgroup	Amino Acids	Molecular Weight	PI
OebZIP1	Oeu008473.1	chr1	S	145	16375.5	6.11
OebZIP2	Oeu006053.1	chr1	I	347	38113.09	5.96
OebZIP3	Oeu051610.1	chr1	D	290	32766.28	8.88
OebZIP4	Oeu056890.1	chr2	A	99	11621.62	10.19
OebZIP5	Oeu022973.2	chr2	D	441	49186.14	6.58
OebZIP6	Oeu022988.1	chr2	B	772	84314.52	6.04
OebZIP7	Oeu006685.1	chr3	F	303	33507.34	6
OebZIP8	Oeu006686.1	chr3	F	288	31203.64	6.26
OebZIP9	Oeu007291.1	chr3	A	424	45839.44	9.53
OebZIP10	Oeu023417.1	chr3	E	360	40341.49	6.68
OebZIP11	Oeu038442.2	chr3	C	472	51398.17	6.59
OebZIP12	Oeu002963.1	chr3	I	537	59000.57	6.36
OebZIP13	Oeu063654.1	chr3	G	318	35242.88	9.11
OebZIP14	Oeu023263.1	chr4	A	274	29798.8	8.37
OebZIP15	Oeu024789.3	chr4	D	369	42321.12	8.28
OebZIP16	Oeu040315.1	chr4	I	248	27513.78	7.06
OebZIP17	Oeu052980.1	chr5	S	149	17126.38	6.59
OebZIP18	Oeu020739.1	chr5	S	159	18088.17	7.9
OebZIP19	Oeu000648.1	chr5	A	274	28952.18	5.04
OebZIP20	Oeu037315.1	chr7	S	138	16462.85	9.71
OebZIP21	Oeu064765.1	chr7	I	401	43971.77	5.75
OebZIP22	Oeu038190.1	chr7	D	391	44031.76	5.48
OebZIP23	Oeu040621.1	chr7	S	157	18560.67	6.07
OebZIP24	Oeu012566.1	chr7	A	191	21281.87	9.98
OebZIP25	Oeu024140.1	chr8	S	176	20683.12	5.18
OebZIP26	Oeu042050.1	chr8	D	451	49267.32	6.71
OebZIP27	Oeu059352.1	chr9	D	459	50759.51	7.23
OebZIP28	Oeu056908.1	chr9	J	457	50704.33	9.18
OebZIP29	Oeu062195.1	chr10	S	163	19314.59	5.66
OebZIP30	Oeu037894.1	chr10	G	260	27321.18	9.14
OebZIP31	Oeu046251.1	chr10	I	474	52633.26	6.05
OebZIP32	Oeu049987.2	chr10	A	426	45662.1	9.47
OebZIP33	Oeu002037.1	chr10	S	162	18996.33	5.2
OebZIP34	Oeu005395.1	chr10	E	291	33007.88	6.68
OebZIP35	Oeu022456.1	chr11	I	170	19366.65	6.59
OebZIP36	Oeu009222.1	chr11	A	358	39999.46	7.13
OebZIP37	Oeu038018.1	chr12	S	145	16445.55	5.91
OebZIP38	Oeu054419.1	chr12	I	350	38367.21	6.01
OebZIP39	Oeu023589.1	chr13	G	336	36700.88	6.62
OebZIP40	Oeu020468.3	chr13	D	404	45538.76	7.79
OebZIP41	Oeu012420.3	chr14	A	486	52207.15	8.8
OebZIP42	Oeu054712.4	chr14	I	467	51485.75	7.63
OebZIP43	Oeu024578.1	chr14	D	359	40535.04	6.42
OebZIP44	Oeu030419.1	chr14	S	169	19111.5	9.1

OebZIP45	Oeu049343.1	chr14	S	167	19891.12	5.96
OebZIP46	Oeu055647.2	chr15	I	541	58955.45	8.85
OebZIP47	Oeu049463.1	chr15	F	246	27219.44	6.08
OebZIP48	Oeu026678.1	chr15	F	292	31832.74	6
OebZIP49	Oeu013716.1	chr15	A	421	45964.71	9.41
OebZIP50	Oeu002658.2	chr16	C	433	46192.2	5.54
OebZIP51	Oeu036581.2	chr16	G	401	44145.36	6.5
OebZIP52	Oeu049833.1	chr16	E	368	41568.85	8.44
OebZIP53	Oeu049834.1	chr16	E	424	47783.19	6.58
OebZIP54	Oeu044311.2	chr16	A	346	37369.78	9.69
OebZIP55	Oeu046151.1	chr16	S	156	17786.89	6.84
OebZIP56	Oeu063787.1	chr17	D	440	48906.61	6.4
OebZIP57	Oeu033749.1	chr17	D	490	54762.37	6.48
OebZIP58	Oeu014896.2	chr17	A	421	46923.22	9.18
OebZIP59	Oeu044216.1	chr17	A	304	33809.98	8.98
OebZIP60	Oeu044242.3	chr17	B	722	78729.68	6.07
OebZIP61	Oeu013337.1	chr18	S	216	25141.36	10.35
OebZIP62	Oeu032847.1	chr18	S	178	21070.24	5.73
OebZIP63	Oeu021814.1	chr20	S	186	21531.97	5.68
OebZIP64	Oeu061329.1	chr21	E	286	32290.14	5.88
OebZIP65	Oeu019969.1	chr22	S	146	17849.43	9.17
OebZIP66	Oeu051270.1	chr23	A	203	22385.83	7.83
OebZIP67	Oeu053639.1	scaffold65	S	140	16408.65	7.84
OebZIP68	Oeu027098.1	scaffold227	G	723	75900.89	5.78
OebZIP69	Oeu033274.1	scaffold279	E	308	34055.14	6.07
OebZIP70	Oeu034446.1	scaffold290	I	501	55691.82	6.31
OebZIP71	Oeu039193.1	scaffold349	A	231	26361	6.54
OebZIP72	Oeu042496.1	scaffold402	K	475	53523.54	5.29
OebZIP73	Oeu042677.1	scaffold406	H	146	16110.01	9.8
OebZIP74	Oeu047760.1	scaffold502	S	149	16580.37	9.1
OebZIP75	Oeu047761.1	scaffold502	S	149	16580.37	9.1
OebZIP76	Oeu048005.1	scaffold508	C	324	35395.44	5.53
OebZIP77	Oeu061222.1	scaffold864	S	156	17519.61	6.3
OebZIP78	Oeu061400.1	scaffold870	F	253	27766.94	5.73
OebZIP79	Oeu062531.2	scaffold910	A	390	44456.53	5.48
OebZIP80	Oeu063651.2	scaffold948	D	392	43767.24	8.83
OebZIP81	Oeu064834.1	scaffold996	E	273	30824.1	5.7
OebZIP82	Oeu002120.1	scaffold1069	D	361	41080.35	5.77
OebZIP83	Oeu002378.1	scaffold1075	D	516	57560.22	9.39
OebZIP84	Oeu007032.1	scaffold1259	S	135	15654.66	8.86
OebZIP85	Oeu009250.1	scaffold1344	E	307	33906.98	5.87
OebZIP86	Oeu016439.1	scaffold1661	C	430	47086.61	4.88
OebZIP87	Oeu022850.1	scaffold2010	G	366	39710.23	7.73
OebZIP88	Oeu024201.1	scaffold2091	H	151	16646.58	9.74
OebZIP89	Oeu024476.1	scaffold2105	I	416	45718.1	6.19
OebZIP90	Oeu025468.1	scaffold2179	I	329	36355.23	6.81
OebZIP91	Oeu027468.1	scaffold2299	C	396	42244.74	6.15
OebZIP92	Oeu030595.1	scaffold2544	S	173	19519.88	5.44
OebZIP93	Oeu030596.1	scaffold2544	S	173	19519.88	5.44
OebZIP94	Oeu031080.1	scaffold2586	G	339	35794.44	8.47
OebZIP95	Oeu031096.1	scaffold2588	A	265	28842.65	5.78

OebZIP96	Oeu032433.1	scaffold2701	S	199	23151.24	10.99
OebZIP97	Oeu032649.1	scaffold2724	A	279	29589.64	4.85
OebZIP98	Oeu033308.1	scaffold2792	S	198	23016.1	9.6
OebZIP99	Oeu034905.1	scaffold2953	A	96	11219.82	10.06
OebZIP100	Oeu035037.1	scaffold2971	I	223	25096.09	6.76
OebZIP101	Oeu038305.1	scaffold3364	C	329	36594.37	5.2
OebZIP102	Oeu039077.1	scaffold3462	D	483	54002.69	6.53
OebZIP103	Oeu020389.1	scaffold18833	A	183	20394.07	9.51

Table 2S. The chromosomal location of olive *LPG* genes

Gene name	Sequence ID	Chromosome	Location	Location
OeACC.1	Oeu028727.1	chr11	34160138	34164781
OeACC.2	Oeu040801.1	scaffold374	349452	356089
OeBCCP.5	Oeu027146.1	scaffold2272	169866	172955
OeBCCP.4	Oeu008266.1	scaffold1300	359914	363380
OeBCCP.1	Oeu009735.1	chr12	6090663	6093260
OeBCCP.3	Oeu010163.1	scaffold139	197050	200739
OeBCCP.2	Oeu063805.1	chr12	8943333	8949827
OeSMT	Oeu025052.1	scaffold2149	39357	40500
OeKASIII.2	Oeu056513.1	scaffold724	258679	263272
OeKASIII.1	Oeu002545.1	chr10	39428843	39435183
OeKASIII.4	Oeu009849.2	scaffold1374	156077	166073
OeKASIII.3	Oeu056512.1	scaffold724	251022	252042
OeKASI.5	Oeu040110.1	scaffold363	461114	465082
OeKASI.3	Oeu030911.1	chr21	16503822	16507640
OeKASI.1	Oeu052382.1	chr7	14849274	14853149
OeKASI.2	Oeu042441.1	chr10	40230736	40236627
OeKASI.4	Oeu058007.1	chr22	12649110	12652076
OeKASII.4	Oeu010830.1	chr11	29969801	29982637
OeKASII.5	Oeu043936.2	chr13	10472613	10482152
OeKASII.6	Oeu000294.1	chr17	4399695	4408495
OeKASII.7	Oeu042061.1	scaffold39611	254	1035
OeKASII.3	Oeu008522.1	chr10	32219479	32223528
OeKASII.1	Oeu055336.2	chr2	14774844	14782369
OeKASII.2	Oeu007428.1	chr2	22644153	22645690
OeSACPD.5	Oeu009943.1	scaffold13793	4	2912
OeSACPD.4	Oeu015714.1	chr22	3938331	3939457
OeSACPD.6	Oeu024466.1	scaffold2105	97	1559
OeSACPD.2	Oeu025716.1	chr7	5843823	5845598
OeSACPD.3	Oeu040504.1	chr10	37933279	37937683
OeSACPD.7	Oeu048444.1	scaffold5181	14310	15402
OeSACPD.1	Oeu050331.1	chr4	9838998	9841967
OeFAD2.2	Oeu013924.1	chr4	15922863	15926554
OeFAD2.5	Oeu058547.1	chr22	3270497	3273603
OeFAD2.4	Oeu033739.1	chr17	709438	713180
OeFAD2.3	Oeu007766.1	chr9	12564973	12569165
OeFAD2.1	Oeu061755.1	chr3	23367563	23369376
OeFAD3.2	Oeu004670.1	scaffold1172	93747	97057
OeFAD3.1	Oeu015599.1	chr10	14479754	14482452

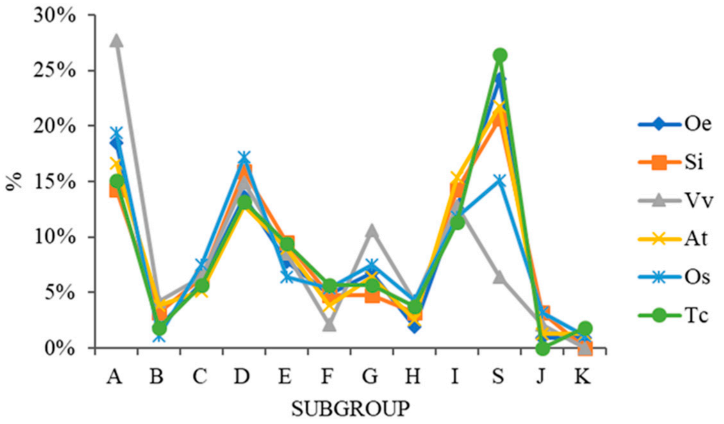

Figure 1S. Classification of *bZIP* genes in six plants. All of *bZIP* genes from different species are constructed with Arabidopsis by the ML method and divided into 12 subgroups (A~K, S). Oe represents olive; Si represents sesame; Vv represents grape; At represents Arabidopsis; Os represents rice and Tc represents cacao.

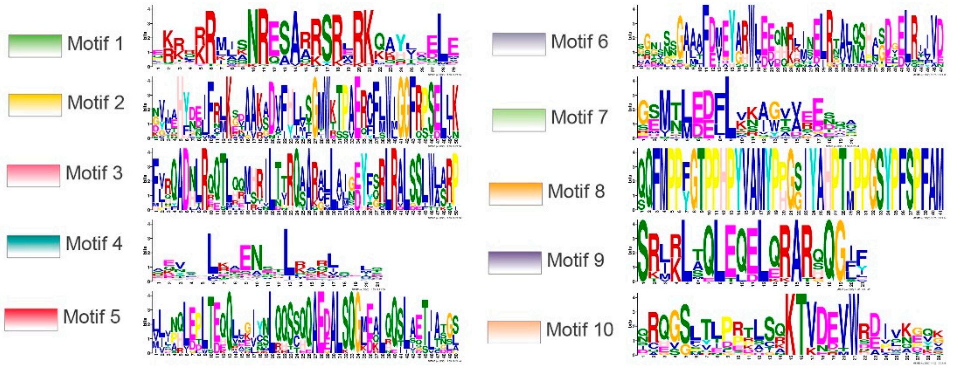

Figure 2S. A detailed motif introduction for all OebZIP proteins.

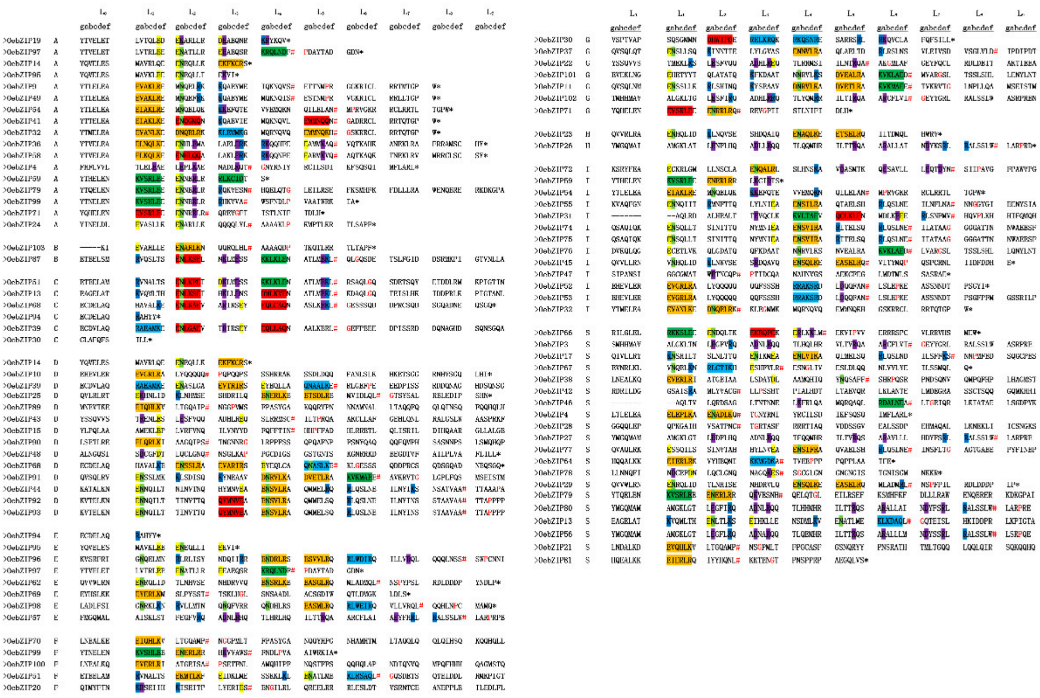

Figure 3S. Amino acid sequences of the Leu zipper region of OebZIP proteins. OebZIP proteins are classified into 12 classes (A~K, S) and some subgroups have similar predictive dimerization

characteristics. The leucine zipper region is divided into heptads (*gabcdef*) from L_0 to L_9 potential visualized *g*↔*e′* pairs. Four colors are used to distinguish different *g*↔*e′* pairs. The attractive basic-acidic pair (R↔E, K↔E) is green, and the attractive acid-basic double (E↔R, E↔K, D↔R and D↔K) is orange, repulsive the basic pairs (K↔K, R↔K, R↔Q, Q↔K and K↔Q) are blue and the disgusting acid pairs (E↔E, E↔D, E↔Q and Q↔E) are red. If a single amino acid at the *e* or *g* position is charged, the residue is dark blue for basic amino acids and red for acidic amino acids. If *a* or *d* is charged, it is purple. The *a*-position of asparagine is light green. The bold font, **G** and **P**, represent that these two amino acids have a potential function to stop the α-helix. The predicted c-terminal boundary is represented by the symbol #, instead of the natural terminals which are indicated by symbol *.

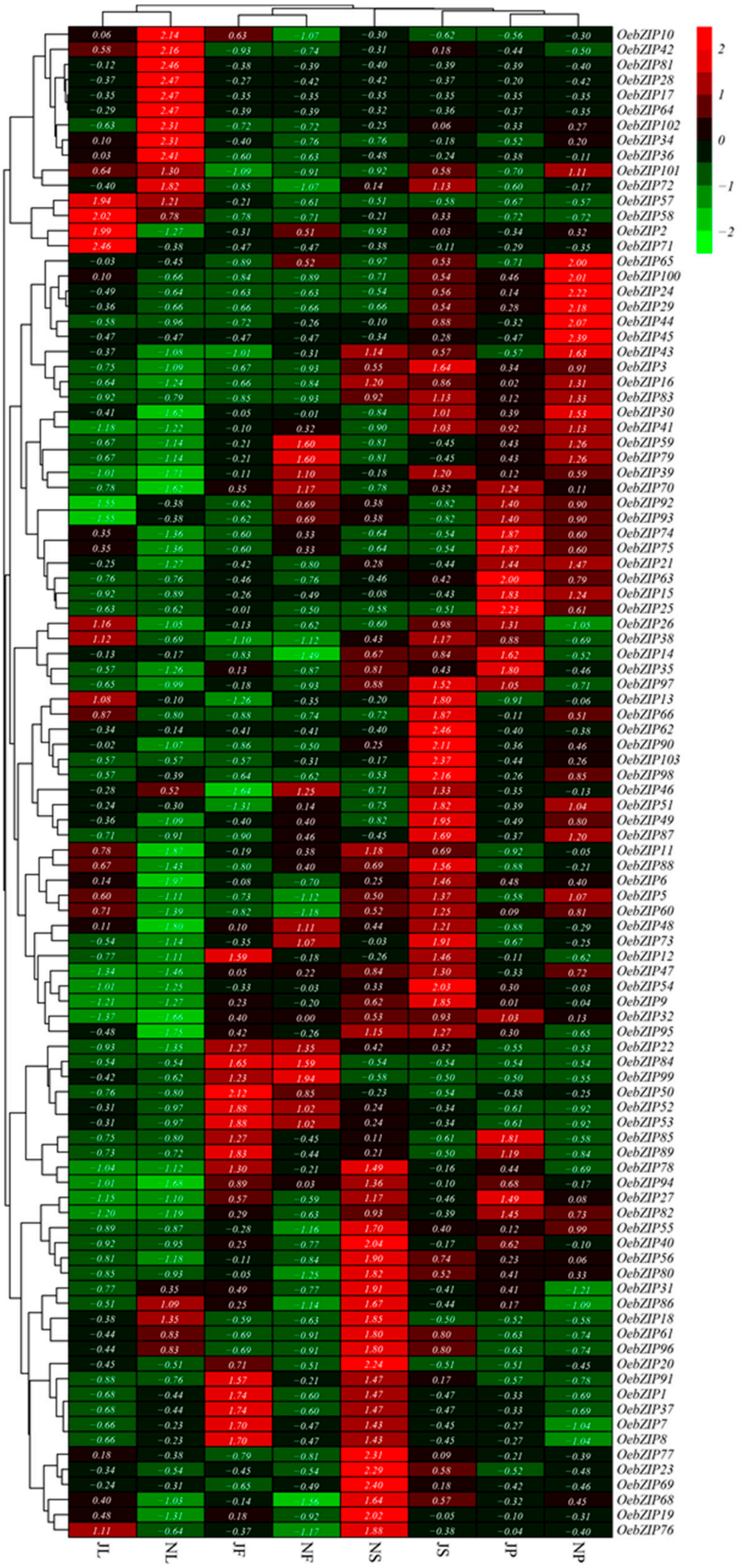

Figure 4S. Heatmap of 103 *OebZIP* genes expressed among four tissues based on transcription data. The P, L, F and S denote pedicel, leaf, fruit and stem, respectively. The J and N mean July and November. Green indicates low expression, dark indicates intermediate expression, and red indicates high expression. The number in the boxes indicate the normalized scale. The heatmap was created using *R*.

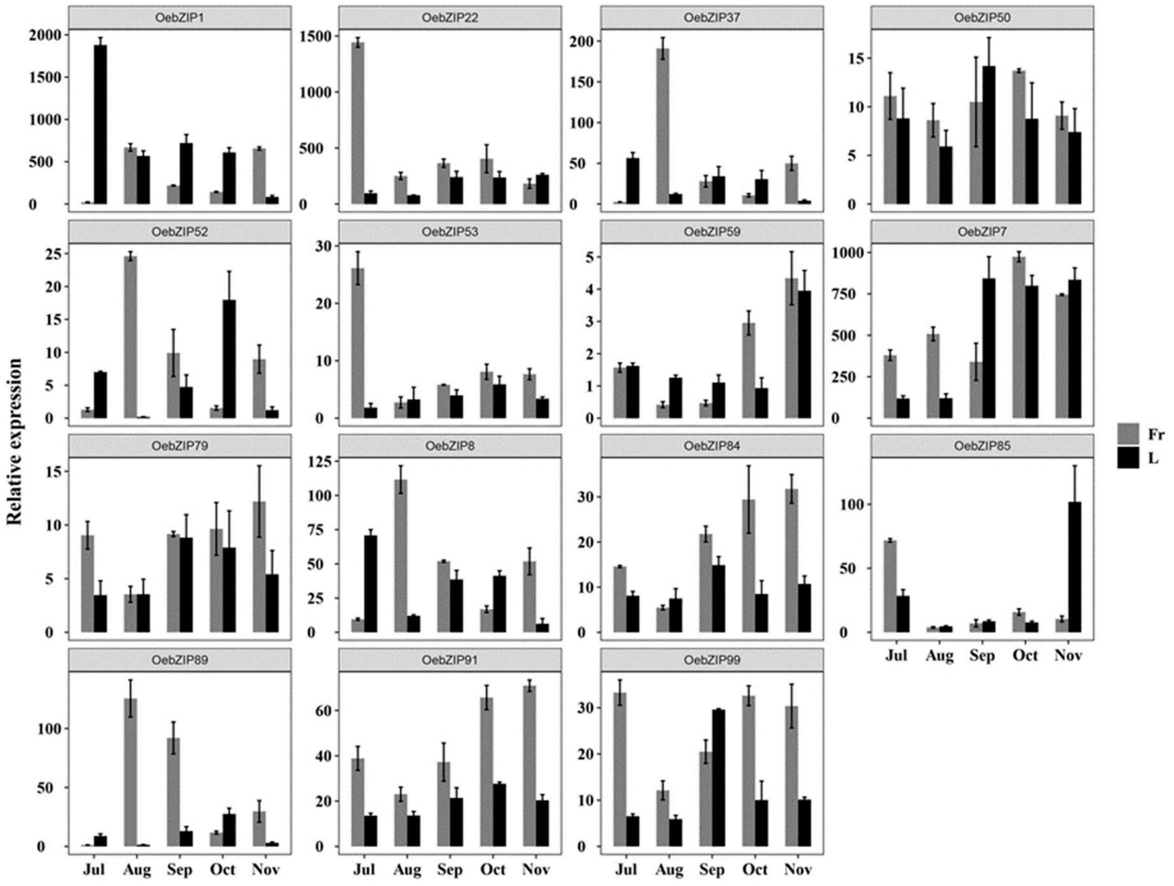

Figure 5S. Relative expression of *OebZIP* genes in fruit and leaf. The expression patterns of 15 olive bZIP genes in fruit and leaf from July to November were examined using a qRT-PCR. The Fr indicates fruit and the L indicates leaf, respectively. The error bars represent the mean values of three replicates ± standard deviation (SD).

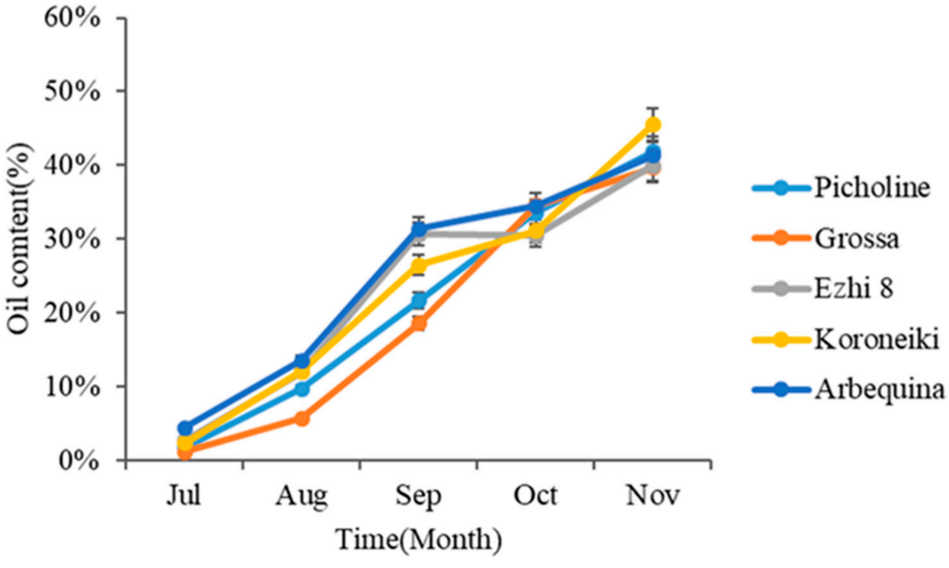

Figure 6S. Dried fruit oil content during the fast oil accumulation stages (July to November).

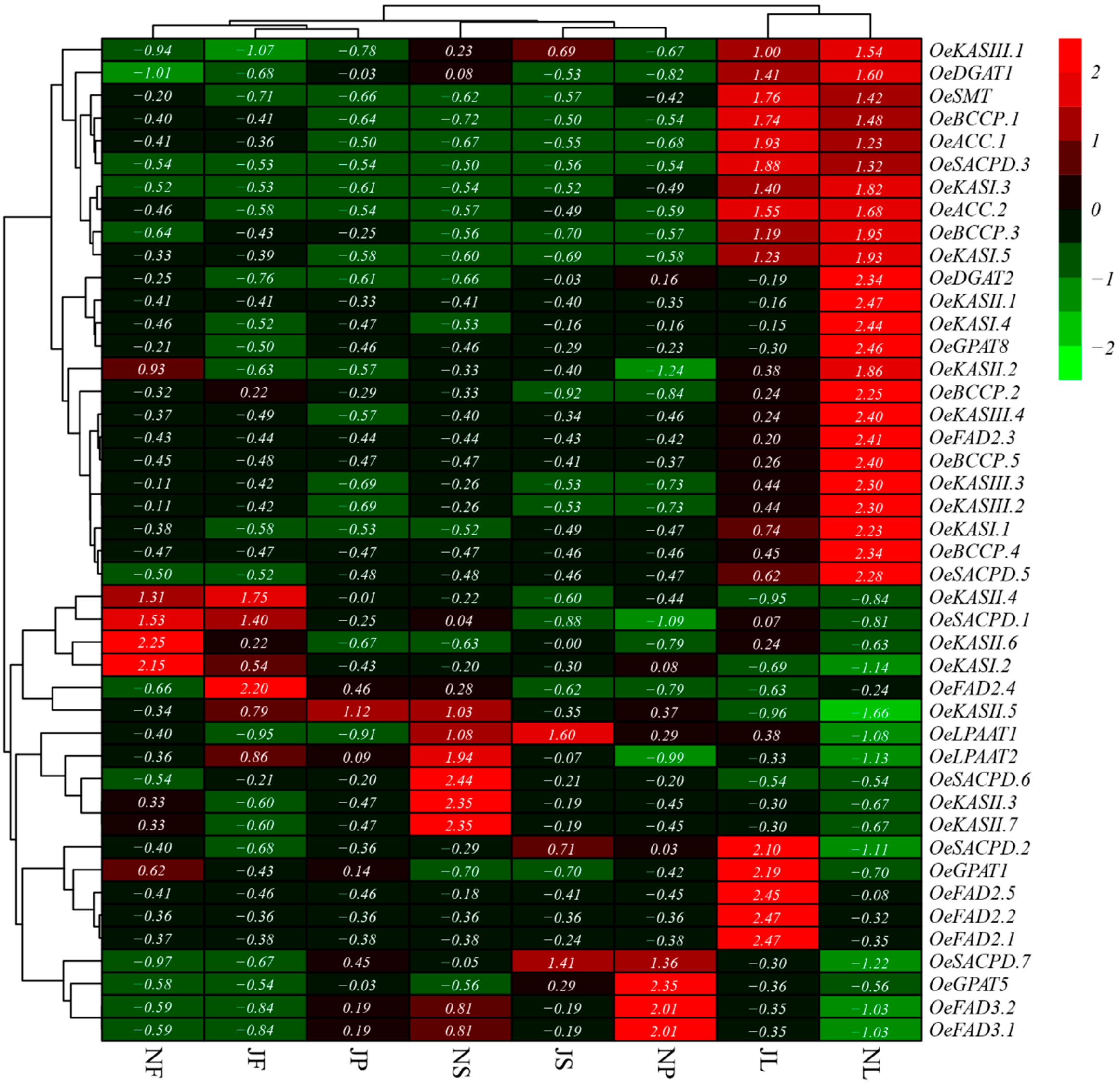

Figure 7S. Heatmap of 44 *LPG* genes expressed among four tissues based on transcription data. The P, L, F and S denote pedicel, leaf, fruit and stem, respectively. The J and N mean July and November. Green indicates low expression, dark indicates intermediate expression, and red indicates high expression. The number in the boxes indicate normalized scale. The heatmap was created using *R*.
